# Screening for *REEP1* Mutations in 31 Chinese Hereditary Spastic Paraplegia Families

**DOI:** 10.3389/fneur.2020.00499

**Published:** 2020-06-23

**Authors:** Xinran Ma, Ji He, Xiaoxuan Liu, Dongsheng Fan

**Affiliations:** ^1^Department of Neurology, Peking University Third Hospital, Beijing, China; ^2^Beijing Municipal Key Laboratory of Biomarker and Translational Research in Neurodegenerative Diseases, Beijing, China; ^3^Key Laboratory for Neuroscience, National Health Commission/Ministry of Education, Peking University, Beijing, China

**Keywords:** hereditary spastic paraplegia, receptor expression-enhancing protein 1, mutation analysis, transethnic, hotspot

## Abstract

**Background:** REEP1 is a common cause of autosomal dominant hereditary spastic paraplegia (HSP) but is rare in China. The pathological mechanism of REEP1 is not fully understood.

**Methods:** We screened for *REEP1* mutations in 31 unrelated probands from Chinese HSP families using next-generation sequencing targeting pathogenic genes for HSP and other related diseases. All variants were validated by Sanger sequencing. The proband family members were also screened for variants for the segregation analysis. All previously reported *REEP1* mutations and cases were reviewed to clarify the genetic and clinical features of REEP1-related HSP.

**Results:** A pathogenic mutation, *REEP1*c. 125G>A (p.Trp42^*^), was detected in a pure HSP family from North China out of 31 HSP families (1/31). This locus, which is located in the second hydrophobic domain of REEP1, is detected in both Caucasian patients with complicated HSP phenotypes and Chinese pure HSP families.

**Conclusion:** REEP1-related HSP can be found in the Chinese population. The 42nd residue is a novel transethnic mutation hotspot. Mutations in this spot can lead to both complicated and pure form of HSP. Identification of transethnic hotspot will contribute to clarify the underlying pathological mechanisms.

## Introduction

Hereditary spastic paraplegia (HSP) comprises a group of neurodegenerative diseases characterized by spastic paraplegia of the lower limbs (1). Hereditary spastic paraplegia is classified as pure or complicated HSP based on whether impairment is restricted to the pyramidal system (2). Approximately 79 pathogenic genes for HSPs have been found (3). These diseases can be inherited in various ways, including autosomal dominant (AD), recessive, X-linked, mitochondrial, and other mechanisms (4). The only treatment to date for HSP is symptomatic treatment. Because HSPs are monogenic diseases, gene therapies, and precision medicine may be appropriate (3).

Loss-of-function mutations of REEP1 (receptor expression enhancing protein 1), a mediator of endoplasmic reticulum (ER)–mitochondrial interactions, can lead to AD HSP (5–7). In a previous study of HSP cohorts, *REEP1* mutations were found to be rare in the Chinese population (8). Here, we screened for *REEP1* mutations using next-generation sequencing (NGS) in 31 Chinese HSP families and performed a general review of REEP1-related HSP, which helped to elucidate the genetic and clinical features of this disease.

## Methods

### Subjects

From January 2012 to September 2019, 31 Chinese families clinically diagnosed with HSP according to Harding's criteria (2) in Peking University Third Hospital were enrolled in this study. All the probands and their relatives received detailed clinical examinations. All participants provided written informed consent. The study was approved by the Peking University Third Hospital ethics committee.

### Genetic Test and Mutation Analysis

Peripheral blood was obtained from all the participants, and DNA was isolated. Next-generation sequencing targeting ~160 genes related to Charcot-Marie-Tooth disease, HSP, and amyotrophic lateral sclerosis, including *REEP1*(NM_022912.2), was conducted (the gene list and detailed sequencing and mutation analysis procedure are shown in Supplementary File and Supplementary Table 1). All identified variants were validated by Sanger sequencing. The relatives of the probands were also screened for these variants via Sanger sequencing for the segregation analysis. The detailed Sanger sequencing procedure for the identified *REEP1* variants is shown in Supplementary Table 2.

## Results

Thirty-one unrelated HSP probands and their relatives from mainland China were recruited for the study (Table 1). Twenty-one probands were male, and 10 were female. The average age at onset was 33.8 ± 13.3 years. Ten families presented with a complicated phenotype. The accompanying symptoms included neuropathy (5/10), extrapyramidal impairments (parkinsonism 1/10, dystonia 1/10), white matter lesions (1/10), dysphagia (1/10), deafness (1/10), nystagmus (1/10), and cognitive impairment (1/10).

**Table 1:** Clinical features of the HSP cohort in this study.

**Total**	**31**
Sex: male/female	21/10
Age at onset of the probands (mean ± SD)	33.8 ± 13.3 years
Phenotypes (complicated)	10 (32.3%)
Polyneuropathy	5
Extra pyramidal signs(parkinsonism, dystonia)	2
White matter lesion	1
Dysphagia	1
Deafness	1
Nystagmus	1
Cognitive impairment	1
Pure	21 (67.7%)

### Genetic Results

**Figure 1:**
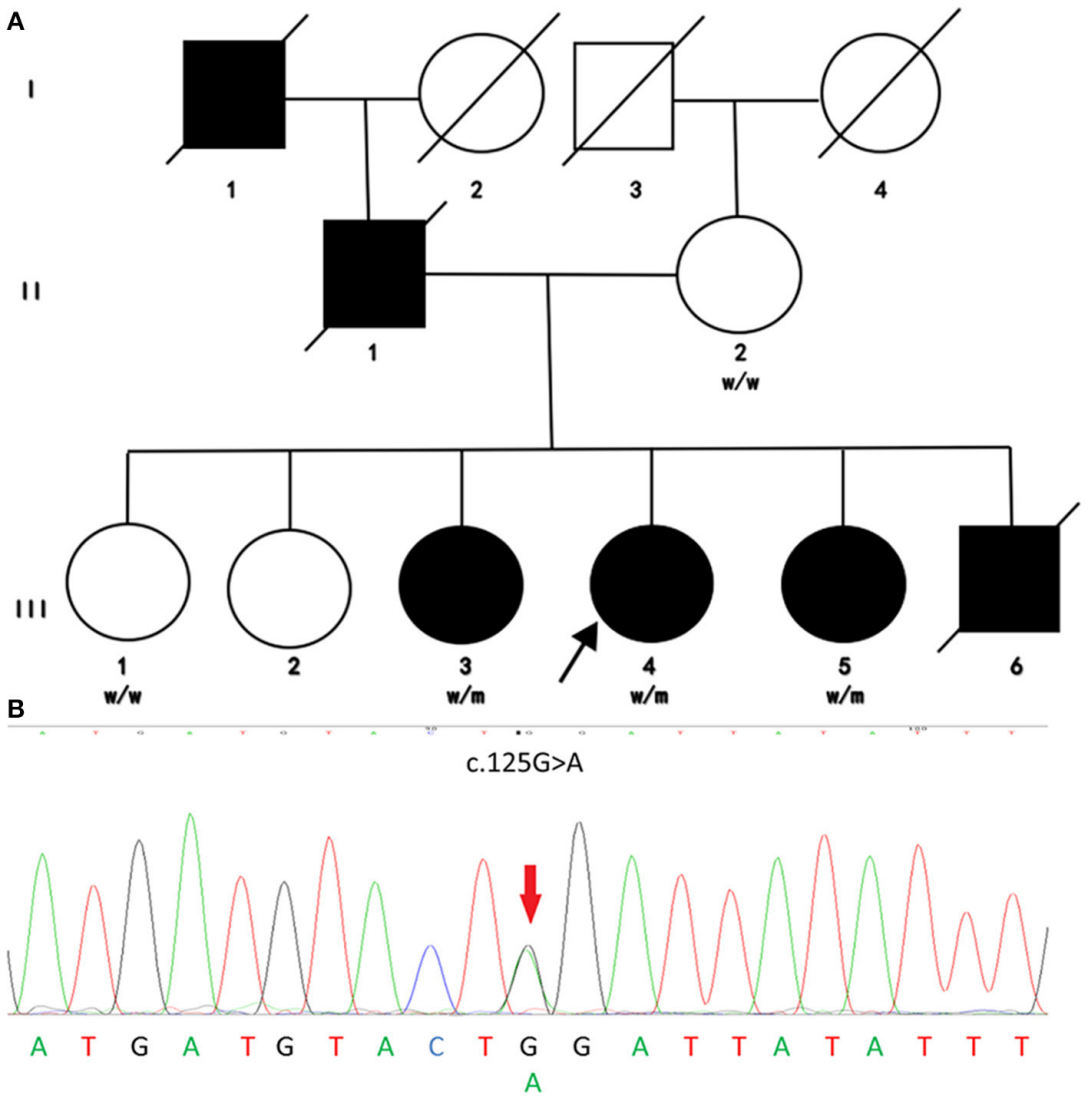
Family pedigree and genomic sequencing electropherograms of the investigated HSP family. **(A)** Pedigree of the investigated family. Males and females are represented by squares and circles, respectively, and filled and unfilled symbols represent affected and unaffected individuals, respectively. The crossed symbols indicate deceased individuals. W, wild type; M, mutated. **(B)** Genomic sequencing electropherograms. The c.125G>A(p.Trp42*) heterozygous non-sense mutation of REEP1 was detected in this HSP family. This mutation cosegregated with an early-onset pure HSP phenotype, supporting the notion that this mutation is pathogenic for HSP.

Genetic variants in pathogenic genes of HSP were identified in eight probands, with a diagnostic rate of 25.8%. Three of them were known causative mutations for HSP (Table 2) (9–11). Two known pathogenic mutations and a novel mutation of *SPAST* were detected in three probands (9.7%). The possible damaging variants were listed in Supplementary Table 3, including *KIAA0196* (1/31), *AP5Z1* (1/31), *DDHD1* (1/31), and *SPG7* (1/31). A previously reported (9) pathogenic non-sense mutation of *REEP1* c. 125G>A (p.Trp42^*^) (RefSeq NM_022912) in exon 3 was detected in a pure HSP proband via NGS and then validated by Sanger sequencing (Figure 1).

**Table 2:** Pathogenic and likely pathogenic mutations of *REEP1* and *SPAST* detected in the HSP cohort.

**Gene**	**Nucleotide change**	**Amino acid change**	**Function prediction**	**Frequency in population database**	**Reference**	**Pathogenicity**
*REEP1*	c. 125G>A	p.Trp42*	Disease-causing	0	(9)	Pathogenic (PVS1,PM1,PP1-PP5)
*SPAST*	c.1664A>G	p.Asp555Gly	Deleterious/probably damaging/disease-causing	0	(10)	Likely pathogenic (PM2, PP1-5)
*SPAST*	c.1176dupT	p.Lys393*	Deleterious/probably damaging/disease-causing	0	(11)	Pathogenic (PVS1, PM1,2,4, PP1-5)

*REEP1, receptor expression-enhancing protein 1; SPAST, spastin; PVS, very strong evidence of pathogenicity; PM, moderate evidence of pathogenicity; PP, supporting evidence of pathogenicity. Functional prediction was made by PolyPhen-2 (http://genetics.bwh.harvard.edu/pph2), SIFT-2 (http://sift.jcvi.org) and Mutation Taster (http://mutationtaster.org). Other unreported possible pathogenic mutations were not shown in this table*.

This mutation was detected in all affected members but not the unaffected ones via Sanger sequencing, which is consistent with an AD model of inheritance. Moreover, the mutation was not found in population databases such as ExAC and 1,000 Genomes. Obvious cosegregation was found in the examined family. Next-generation sequencing showed no pathogenic or likely pathogenic mutations in other causative genes of spastic paraplegia such as *SPAST* and so on. The phenotype of these individuals was also consistent with a previous case. Therefore, we concluded that *REEP1* c.125G>A (p.Trp42^*^) is a pathogenic mutation in this family.

### Clinical Manifestation of the HSP Family With REEP1 Mutation

All the cases in the family from North China with the mutation were consistent with pure HSP (Figure 1B). The proband (III-4) was a 51-year-old woman who complained of walking difficulty and lower limb stiffness starting at ~20 years of age. Recently, she had also experienced urgency of urination without urinary incontinence. Her symptoms progressed slowly during subsequent years. Her family members (I-1, II-1, III-3, III-5, and III-6) had similar symptoms that were limited to lower limb stiffness and urgency of urination. They all received detailed clinical examinations. Other systems were normal. The age at onset for III-3, III-4, III-5, and III-6 ranged from 10 to 30 years. Patient III-6 died of a traffic accident when he was 31 years old.

## Discussion

In this study, genetic variants in HSP genes were detected in eight probands. The frequencies of rare HSP genes in our study are similar to those in a previous study (12). Although *SPAST* was the most common cause for ADHSP (12), accounting for ~50% of ADHSP families in China (13), known pathogenic *SPAST* mutations were detected in only two probands in our cohort (the detailed information are shown in Supplementary File). That may be due to different sequencing methods and small sample size. (We used NGS-based method to detect copy number variation in Supplementary File). Our study may indicate the advantages and disadvantages of NGS. Other sequencing methods could not be substituted.

A known pathogenic mutation, p.W42^*^, in the second hydrophobic domain (HD) of REEP1, was detected, which was previously detected in a pure HSP patient in Norway (9). W42R is a missense mutation in the same amino acid that was found to cause complicated HSP with neuropathy in French Caucasians (14). Both non-sense and missense mutations at the W42 locus have been found to be pathogenic in different ethnicities, indicating that this locus is a transethnic hotspot that plays an important role in the pathogenesis of HSP. Mutations in this locus can lead to both pure and complicated phenotypes, indicating substantial heterogeneity of this transethnic hotspot.

*REEP1* is a causative gene of HSP and distal hereditary motor neuropathy type 5B (15), and REEP1-related diseases also include 2p11.2-2p12 deletion syndrome (16). The extension of the REEP1 protein and mislocalized REEP1 can lead to “toxic gain of function” and result in dHMN (15, 17), whereas loss of function may lead to HSP (5–7).

**Figure 2:**
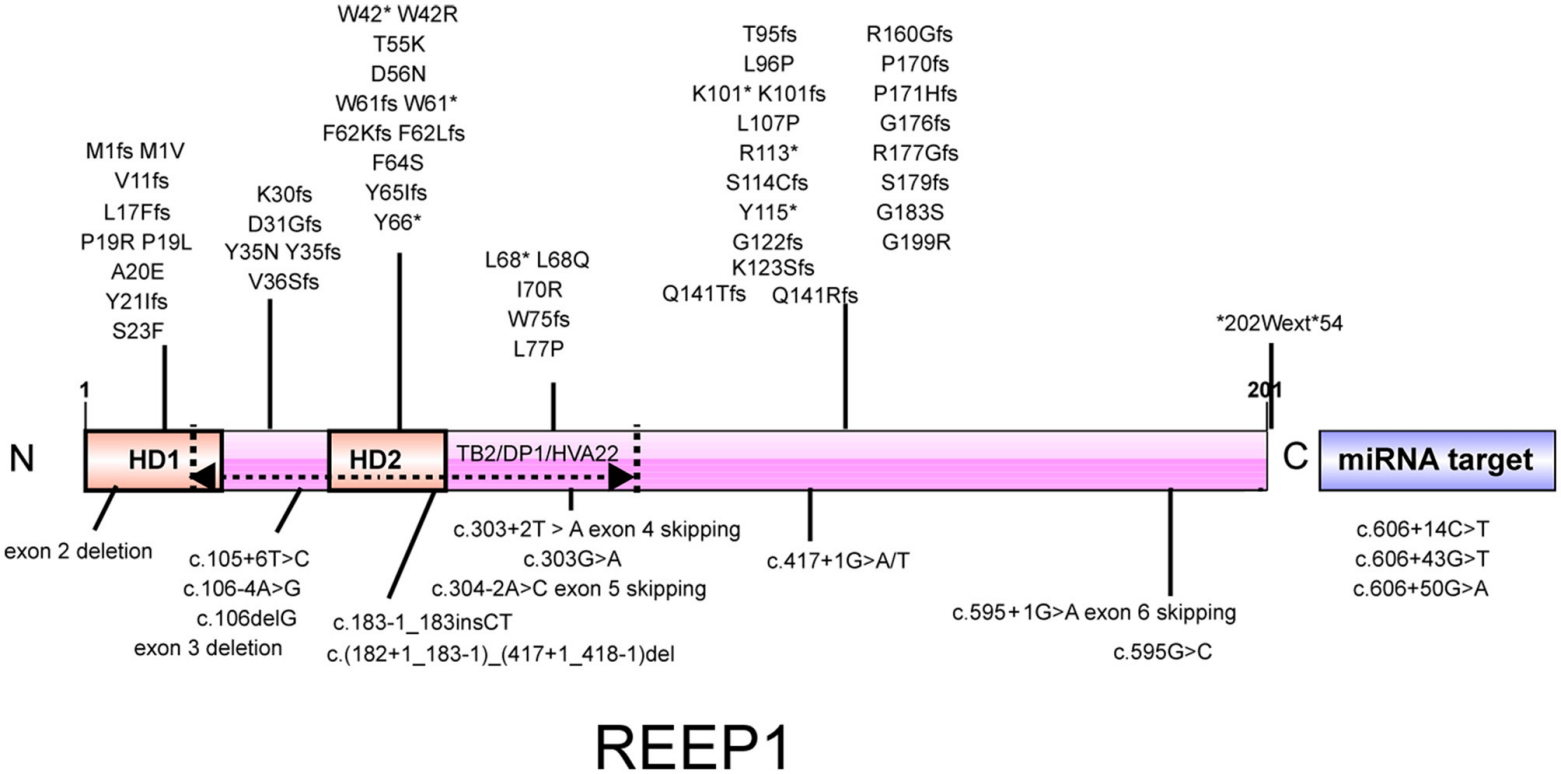
Illustration of the REEP1 protein. REEP1 contains 2 hydrophobic domains and a conserved TB2/DP1/HVA22 domain. There is also a miRNA target region in the 3′ UTR of *REEP1* mRNA. All reported pathogenic mutations of *REEP1* are shown except for 2p11.2-2p12 deletions. HD, hydrophobic domain.

The REEP1 protein is located in the mitochondria and ER and participates in the functional activities of organelles, such as the interaction between the tubular ER and microtubules and peripheral ER shaping (5–7). To date, ~60 pathogenic mutations of REEP1 have been reported, including missense mutations, non-sense mutations, exon deletions, splicing site mutations, and miRNA binding site mutations (Figure 2). The REEP1 protein has a conserved TB2/DP1/HVA22 domain that may have a chaperone-like function (7, 18). Additionally, it contains a mitochondria-localization domain (6) (between aa116 and aa157 in NP_075063.1) and a cytoplasmic C-terminus that is in contact with microtubules (19). There is also a highly conserved miRNA binding site in the 3′ UTR of REEP1 mRNA, and pathogenic mutations in this region influence its post-transcriptional regulation (20). Many missense mutations of REEP1 are located near the N terminus (20, 21), indicating that it is a hotspot region. Mutations in the N terminus (before the 55th amino acid) influence the localization of REEP1 in the ER (21). This region contains two HDs, HD1 in the N terminus, and HD2 near the middle, which is located in the conserved domain. HD2 forms a hairpin-like structure to interact with SPAST and ALT1 in the ER (19, 21). Their interactions mediate ER shaping and are very important for the ER network between the cell body and axon in motor neurons (22). Disruption of the hairpin domain harms the ER organization in distal axons, which may explain the length-dependent degeneration of upper motor neurons in HSP (22). The novel transethnic hotspot W42 is located in the hairpin domain. Thus, it can disturb the normal function of this domain and lead to pathogenesis.

More than 70 REEP1-related HSP pedigrees have been reported (7, 9, 14, 15, 17, 20, 23–35), and their genotypes and phenotypes are summarized in Supplementary Table 4. There is generally an early age at onset, commonly 0–20 and 30–35 years of age (14). The mutation of *REEP1* typically results in AD pure HSP but can cause complicated HSP. The accompanying symptoms include neuropathy (23), tremor, and cognitive impairment (14). Few mutations can lead to both complicated and pure HSP phenotypes. The clinical manifestations can also vary among different ethnicities.

The mutation rate of *REEP1* in HSP varies in different regions (7, 13, 14, 24, 26–29, 31, 36) (Table 3). Although *REEP1* was reported to be the third most common cause of HSP in some countries (20), previous screening studies in Chinese patients did not find pathogenic *REEP1* variants (8, 13). In the present study, we found one family with pathogenic *REEP1* mutation out of 31 HSP families, which is uncommon.

**Table 3:** REEP1 mutation rate in different regions.

**Region**	**Result**	**Reference**
China	0/120 (54 ADHSP families and 66 sporadic cases)	(13)
	1/31 (31 HSP families)	Our study
Germany	4.3% ADHSP (162 pure HSP families)	(26)
The United Kingdom	2.3% ADHSP (133 families and 80 cases)	(27)
The Netherlands	7.4% in SPAST negative AD HSP (27 families and 110 cases)	(28)
Europe	6.5% all HSP (90 families)	(7)
France	4.5% ADHSP (175 families)	(14)
North America	5.0% all HSP (120 patients)	(29)
Poland	3.2% all HSP (85 families and 131 cases)	(31)
Korea	0/27 (27 patients)	(36)
Japan	4.1% ADHSP (66 families and 63 cases)	(24)

*ADHSP, autosomal dominant hereditary spastic paraplegia; SPAST, spastin*.

## Conclusion

REEP1-related HSP can be found in the Chinese population. The 42nd residue is a novel transethnic mutation hotspot. Mutations in this spot can lead to both complicated and pure form of HSP. Identification of transethnic hotspot will contribute to clarify the underlying pathological mechanisms.

## Data Availability Statement

The datasets generated for this study are available on request to the corresponding author.

## Ethics Statement

The studies involving human participants were reviewed and approved by the Peking University Third Hospital Ethics Committee. The patients/participants provided their written informed consent to participate in this study.

## Author Contributions

DF conceived this study and provide financial support. XM and JH performed the experiments, analyzed the data, and wrote the manuscript. XL provided supplementary data.

## Conflict of Interest

The authors declare that the research was conducted in the absence of any commercial or financial relationships that could be construed as a potential conflict of interest.
