# Supplementary material for: Screening for REEP1 Mutations in 31 Chinese Hereditary Spastic Paraplegia Families
Source: Front Neurol. 2020 Jun 23;11:499. doi: 10.3389/fneur.2020.00499 (PMC7325443; doi:10.3389/fneur.2020.00499)
Supplement: Supplementary file 1 [file Data_Sheet_1.docx]

Supplementary Material

# Next-generation sequencing:

Genomic DNA was extracted from the blood sample using a DNA isolation kit(Qiagen,512206, Hilden, Germany). Genomic DNA was fragmented into 150-300 bp pieces by an ultrasonic processor (Covaris Acoustic System, Covaris, MA, USA). Then, the fragments were processed by end-repairing, A-tailing and adaptor ligation. 4-cycle pre-capture PCR was used for DNA amplification. Probes from the Agilent and Agilent Sure Select target enrichment system (Agilent Technologies, Santa Clara, CA, USA) were used to capture the target sequences. The final products were sequenced as 150-bp paired-end reads on an Illumina NextSeq 500 platform (Illumina, San Diego, CA, USA). The average read depth is 100**×**. Raw data were filtered and aligned to the human reference genome (hg19) using the BWA Aligner (v0.7.15).

# Pathogenicity analysis of variants:

The pathogenicity of the variants was assessed according to the 2013 ACMG guidelines(1). 1000 Chinese healthy controls and population databases including the Exome Sequencing Project (ESP) (http://evs.gs.washington.edu), Exome Aggregation Consortium (ExAC) database (http://exac.broadinstitute.org/), 1000 Genomes Project (1000G) database (http://www.1000genomes.org/) and Genome Aggregation Database (gnomAD) (http://gnomad.broadinstitute.org/) were used to exclude benign polymorphisms (minor allele frequency ≥ 1%). *In silica* prediction was conducted using PolyPhen-2 (http://genetics.bwh.harvard.edu/pph2), SIFT-2 (http://sift.jcvi.org) and Mutation Taster (http://mutationtaster.org). The conservation of each locus was evaluated by the genomic evolutionary rate profiling (GERP) scores (http://mendel.stanford.edu/SidowLab/downloads/gerp/index.html).

# Copy number variation analysis

Aligned data was transferred into bam format. Copy number variation analysis was conducted via CODEX(2)(<http://www.bioconductor.org/packages/devel/bioc/html/CODEX.html>), XHMM (v 1.0, [https://atgu.mgh.harvard.edu/xhmm/download.shtml](https://atgu.mgh.harvard.edu/xhmm/download.shtml#!/content/playContent/_blank))(3) and CNVkit (https://github.com/etal/cnvkit)(4).

# Sanger sequencing for validation:

The identified REEP1 variant c.125G>A(p.Trp42*) was then validated by Sanger sequencing. The primers are listed in Table 2.

# Supplementary Tables

sTable 1. Gene list for NGS

|  | Gene symbol |
| --- | --- |
| Clinical phenotype: HSP/ALS associated | ALS2, ANG, APEX1, ARHGEF28, CHGB, CHMP2B, CHRNA4, DAO, DCTN1, DIAPH3, DPYSL3, ELP3, ERBB4, EWSR1, FIG4, FUS, GRN, HFE, HNRNPA1, HNRNPA2B1, ITPR2, MAPT, NEFH,OGG1, OPTN, PCP4, PFN1, PRPH, RAMP3, SETX, SIGMAR1, SLC52A2, SLC52A3, SOD1, SOX5, SQSTM1, SS18L1, SUSD1, SYT9, TAF15, TARDBP, TRPM2, UBQLN1, UBQLN2, VAPB, VCP, VEGFA, ZFP64, ZNF512B |
| Clinical phenotype: HSP/CMT associated | AARS,ABCD1, AIFM1, AMPD2, AP4B1, AP4E1, AP4M1, AP4S1, AP5Z1, ARL6IP1, ARSI, ATL1, ATP7A, ATRX, B4GALNT1, BICD2, BSCL2, C12orf65, C19orf12, CCT5, CTDP1, CYP2U1, CYP7B1, DDHD1, DDHD2, DHTKD1, DNAJB2, DNM2, DNMT1, DYNC1H1, EGR2, ENTPD1, ERLIN1, ERLIN2, FA2H, FAM134B, FBLN5, FGD4, FLRT1, GARS, GBA2, GDAP1, GJB1, GJC2, GNB4, HARS, HINT1, HK1, HOXD10, HSPB1, HSPB3, HSPB8, HSPD1, IGHMBP2, IKBKAP, INF2, KARS, KIAA0196, KIF1A, KIF1B , KIF1C, KIF5A, L1CAM, LITAF, LMNA, LRSAM1, MARS, MED25, MFN2, MPZ, MTMR2, MYH14, NDRG1, NEFL, NIPA1, NT5C2, NTRK1, OPA1, PDK3, PGAP1, PLEKHG5, PLP1, PMP22, PNPLA6, PRNP, PRPS1, PRX, RAB3GAP2, RAB7A, REEP1, RTN2, SACS, SBF1, SBF2, SCN9A, SH3TC2, SLC16A2, SLC33A1, SLC5A7, SPG20, SPAST, SPG11, SPG21, SPG7, SPTLC1, SPTLC2, TFG, TRIM2, TRPV4, USP8, WDR48, WNK1, YARS, ZFR, ZFYVE26, ZFYVE27 |

Approximately 160 genes were analyzed (past quality control).

sTable 2. Primers and procedure for Sanger sequencing.

| *REEP1* | chr2:86491145 |  |  |
| --- | --- | --- | --- |
| 5' primer | CTGGCCTGAGGTGAGGTTTG |  |  |
| 3' primer | TCTTCCTGGTGTTGGGCAGT |  |  |
| Conditions for PCR |  |  |  |
| step1 | 95℃ | 10min |  |
| step2 | 95℃ | 30s |  |
| step3 | 60℃ | 30s |  |
| step4 | 72℃ | 45s | to step2 35cycles |
| step5 | 72℃ | 5min |  |

sTable 3. Rare variants in causative genes of HSP detected in the cohort.

| Gene/  RefSq | Nucleotide change | Amino acid change | Pathogenicity |
| --- | --- | --- | --- |
| *KIAA0196*  NM_014846 | c.1109A>G | p.Asn370Ser | VUS(PM2,PP3) |
| *AP5Z1*  NM_014855 | c.2386C>T | p.Arg796Trp | VUS(PM2,PP3) |
| *AP5Z1* | c.790+6C>T | Splicing site | VUS(PM2) |
| *DDHD1*  NM_001160148 | c.1708C>T | p.Arg570* | Likely pathogenic (PVS1,PM2) |
| *DDHD1* | c.838+16847A>G | Intron | VUS (PM2) |
| *SPG7*  NM_003119 | c.1669G>A | p.Ala557Thr | VUS, (PM2,PM6,BP4) |
| *SPAST*  NM_014946 | \| c.1632C>G \| p.Tyr544Ter \| \| --- \| --- \| | p.Y544* | Pathogenic  (PVS1,PM2, PP3-4) |

Abbreviations: VUS: variants of unknown significance; PVS: very strong evidence of pathogenicity; PM: moderate evidence of pathogenicity; PP: supporting evidence of pathogenicity; BP: supporting evidence of benign impact.

sTable 4. Previously reported REEP1-mutated pedigrees.

| Domain | DNA change | Amino acid change | Reference | Clinical feature |
| --- | --- | --- | --- | --- |
| HD1 | c.-1_2delCAT | p.M1fs | (5). | Pure |
|  | c.1A>G | p.M1V | (6) | Pure |
|  | c.33-?_606+?dup | p.V11fs | (5) | Pure |
|  | c.49delC | p.L17Ffs | (7) | Pure |
|  | c.56 C>G | p.P19R | (8) | Pure |
|  | c.56C>T | p.P19L | (7) | Pure |
|  | c.59C>A | p.A20E | (5) | NA |
|  |  |  | (9) | Complicated with learning difficulty |
|  |  |  | (10) | Pure |
|  |  |  | (11) | Complicated with orthostatic tremor |
|  | c.60delG | p.Y21Ifs*7 | (12) | NA |
|  | c.68C>T | p.S23F | (7) | Pure |
|  | c.87 insA | p.K30fs | (13) | Pure |
|  | c.104_105del | p.Y35fs | (5) | Pure |
|  | c.105+6T>C |  | (9) | Pure |
|  | c.106‐4A>G |  | (7) | Complicated with cerebellar ataxia |
|  | c.106delG |  | (7) | Pure |
|  | c.124T>C | p.W42R | (7) | Complicated with polyneuropathy |
|  | c.125G>A | p.W42* | This study | Pure |
|  | c.125C>T | p.W42* | (14) | Pure |
| HD2 | c.164C>A | p.T55K | (12) | NA |
|  | c.166G>A | p. D56N | (7) | Pure |
|  |  |  | (15) | Pure |
|  | c.182-2A>G | p.W61fs | (9) | Complicated with scoliosis |
|  |  |  | (5) | NA |
|  |  |  | (10) | Pure |
|  | c.183-1_183insCT | p.W61fs | (5) | Pure |
|  | c.183-2A>G | p.W61fs | (8) | Pure |
|  |  |  | (7) | Pure |
|  | c.183_184insCT | p.F62Lfs*8 | (12) | NA |
|  | c.193delT | p.Y65Ifs | (5) | NA |
|  | c.198T>G | p.Y66* | (7) | Complicated with cerebellar ataxia and neuropathy |
|  | c.202C>T; c.203T>A | p.L68* | (8) | Pure |
|  | c.209T>G | p.I70R | (8) | Pure |
|  | c.222delC | p.W75fs | (5) | NA |
|  | c.230T>C | p.L77P | (16) | Pure |
|  | c.282delC | p.T95fs | (5) | Pure |
|  | c.303G>A | p.K101fs | (5) | Pure |
|  | c.320T>C | p.L107P | (12) | NA |
|  | c.337C | p.R113* | (17) | Pure |
|  |  |  | (18) | Complicated with polyneuropathy, mitochondrial dysfunction |
|  | c.337C>T | p.R113* | (16) | Pure |
|  | c.340_347delAGTTACGA | p.S114Cfs*70 | (12) | NA |
|  | c.345C>A | p.Y115* | (12) | NA |
|  | c.366G>T |  | (7) | Complicated silver syndrome and cognitive impairment |
|  | c.417+1G>A |  | (19) | Pure |
|  |  |  | (5) | Pure |
|  |  |  | (6) | NA |
|  | c.419_420insG | p.Q141Tfs*46 | (12) | NA |
|  | c.478delA | p.R160Gfs*63 | (12) | NA |
|  | c.507delC | p.P170fs | (5) | NA |
|  |  |  | (10) | Pure |
|  | c.512delC | p.P171Hfs*52 | (16) | Complicated with polyneuropathy. |
|  | c.526delG | p.G176fs | (9) | Complicated with scoliosis and mild distal atrophy |
|  |  |  | (5) | NA |
|  |  |  | (10) | Pure |
|  | c.537_540del | p.S179fs*43 | (5) | Pure |
|  | c.595G>C | p.G199R | (5) | Pure |
|  | c.605A > G; c.*14C > T | p.*202Wext*54 | (20) | CMT2,HMN5B |
| miRNA target | c.606 + 14C>T | miRNA target | (5) | NA |
|  | c.606+43G>T | miRNA target | (10) | Pure |
|  |  |  | (8) | Pure |
|  |  |  | (5) | Pure |
|  |  |  | (18) | Complicated with small fiber neuropathy |
|  |  |  | (9) | NA |
|  | c.606+50G>A | miRNA target | (8) | NA |
|  |  |  | (10) | Pure |
|  |  |  | (9) | Complicated with distal sensory neuropathy |
| Exon deletion | exon 2 deletion | exon 2 deletion | (7) | Complicated with postural tremor |
|  | exon 2-5 deletion | exon 2-5 deletion | (21) | Pure |
|  | exon 3 deletion | exon 3 deletion | (7) | Pure |
|  | c.303+2T > A | exon 4 skipping | (22) | Pure, genetic anticipation |
|  | del exon 4-5 | del exon 4-5 | (13) | Pure |
|  | c.304-2A>C | exon 5 skipping | (23) | dHMN |
|  | c.595 + 1G>A | exon 6 skipping | (24) | Pure |

Abbreviations: TM: transmembrane; NA: not applicable. CMT: Charcot-Marie-Tooth disease. dHMN: distal hereditary motor neuropathy.

# Reference

1. Richards S, Aziz N, Bale S, Bick D, Das S, Gastier-Foster J, et al. Standards and guidelines for the interpretation of sequence variants: a joint consensus recommendation of the American College of Medical Genetics and Genomics and the Association for Molecular Pathology. Genet Med. 2015;17(5):405-24. doi:10.1038/gim.2015.30

2. Jiang Y, Oldridge DA, Diskin SJ, Zhang NR. CODEX: a normalization and copy number variation detection method for whole exome sequencing. Nucleic acids research. 2015;43(6):e39. doi: 10.1093/nar/gku1363

3. Fromer M, Moran JL, Chambert K, Banks E, Bergen SE, Ruderfer DM, et al. Discovery and statistical genotyping of copy-number variation from whole-exome sequencing depth. American journal of human genetics. 2012;91(4):597-607. doi: 10.1016/j.ajhg.2012.08.005

4. Talevich E, Shain AH, Botton T, Bastian BC. CNVkit: Genome-Wide Copy Number Detection and Visualization from Targeted DNA Sequencing. PLoS Comput Biol. 2016;12(4):e1004873. doi: 10.1371/journal.pcbi.1004873

5. Beetz C, Schule R, Deconinck T, Tran-Viet KN, Zhu H, Kremer BP, et al. REEP1 mutation spectrum and genotype/phenotype correlation in hereditary spastic paraplegia type 31. Brain. 2008;131(Pt 4):1078-86. doi:10.1093/brain/awn026

6. de Bot ST, Veldink JH, Vermeer S, Mensenkamp AR, Brugman F, Scheffer H, et al. ATL1 and REEP1 mutations in hereditary and sporadic upper motor neuron syndromes. J Neurol. 2013;260(3):869-75. doi:10.1007/s00415-012-6723-z

7. Goizet C, Depienne C, Benard G, Boukhris A, Mundwiller E, Sole G, et al. REEP1 mutations in SPG31: frequency, mutational spectrum, and potential association with mitochondrial morpho-functional dysfunction. Hum Mutat. 2011;32(10):1118-27. doi:10.1002/humu.21542

8. Elert-Dobkowska E, Stepniak I, Krysa W, Rajkiewicz M, Rakowicz M, Sobanska A, et al. Molecular spectrum of the SPAST, ATL1 and REEP1 gene mutations associated with the most common hereditary spastic paraplegias in a group of Polish patients. J Neurol Sci. 2015;359(1-2):35-9. doi: 10.1016/j.jns.2015.10.030

9. Zuchner S, Wang G, Tran-Viet KN, Nance MA, Gaskell PC, Vance JM, et al. Mutations in the novel mitochondrial protein REEP1 cause hereditary spastic paraplegia type 31. Am J Hum Genet. 2006;79(2):365-9. doi:10.1086/505361

10. McCorquodale DS, 3rd, Ozomaro U, Huang J, Montenegro G, Kushman A, Citrigno L, et al. Mutation screening of spastin, atlastin, and REEP1 in hereditary spastic paraplegia. Clin Genet. 2011;79(6):523-30. doi:10.1111/j.1399-0004.2010.01501.x

11. Erro R, Cordivari C, Bhatia KP. SPG31 presenting with orthostatic tremor. Eur J Neurol. 2014;21(4):e34-5. doi:10.1111/ene.12360

12. Schlang KJ, Arning L, Epplen JT, Stemmler S. Autosomal dominant hereditary spastic paraplegia: novel mutations in the REEP1 gene (SPG31). BMC Med Genet. 2008;9:71. doi:10.1186/1471-2350-9-71

13. Ishiura H, Takahashi Y, Hayashi T, Saito K, Furuya H, Watanabe M, et al. Molecular epidemiology and clinical spectrum of hereditary spastic paraplegia in the Japanese population based on comprehensive mutational analyses. J Hum Genet. 2014;59(3):163-72. doi:10.1038/jhg.2013.139

14. Iqbal Z, Rydning SL, Wedding IM, Koht J, Pihlstrom L, Rengmark AH, et al. Targeted high throughput sequencing in hereditary ataxia and spastic paraplegia. Plos One. 2017;12(3):e0174667. doi:10.1371/journal.pone.0174667

15. Polymeris AA, Tessa A, Anagnostopoulou K, Rubegni A, Galatolo D, Dinopoulos A, et al. A series of Greek children with pure hereditary spastic paraplegia: clinical features and genetic findings. J Neurol. 2016;263(8):1604-11. doi:10.1007/s00415-016-8179-z

16. Toft A, Birk S, Ballegaard M, Duno M, Hjermind LE, Nielsen JE, et al. Peripheral neuropathy in hereditary spastic paraplegia caused by REEP1 variants. J Neurol. 2019;266(3):735-44. doi:10.1007/s00415-019-09196-1

17. Park HJ, Lee MJ, Lee JE, Park KD, Choi YC. Pathogenic Variant of REEP1 in a Korean Family with Autosomal-Dominant Hereditary Spastic Paraplegia. J Clin Neurol. 2018;14(2):248-50. doi:10.3988/jcn.2018.14.2.248

18. Hewamadduma C, McDermott C, Kirby J, Grierson A, Panayi M, Dalton A, et al. New pedigrees and novel mutation expand the phenotype of REEP1-associated hereditary spastic paraplegia (HSP). Neurogenetics. 2009;10(2):105-10. doi:10.1007/s10048-008-0163-z

19. Liu SG, Che FY, Heng XY, Li FF, Huang SZ, Lu DG, et al. Clinical and genetic study of a novel mutation in the REEP1 gene. Synapse. 2009;63(3):201-5. doi:10.1002/syn.20602

20. Bock AS, Gunther S, Mohr J, Goldberg LV, Jahic A, Klisch C, et al. A nonstop variant in REEP1 causes peripheral neuropathy by unmasking a 3'UTR-encoded, aggregation-inducing motif. Hum Mutat. 2018;39(2):193-6. doi:10.1002/humu.23369

21. Battini R, Fogli A, Borghetti D, Michelucci A, Perazza S, Baldinotti F, et al. Clinical and genetic findings in a series of Italian children with pure hereditary spastic paraplegia. Eur J Neurol. 2011;18(1):150-7. doi:10.1111/j.1468-1331.2010.03102.x

22. Kamada M, Kawarai T, Miyamoto R, Kawakita R, Tojima Y, Montecchiani C, et al. Spastic paraplegia type 31: A novel REEP1 splice site donor variant and expansion of the phenotype variability. Parkinsonism Relat Disord. 2018;46:79-83. doi:10.1016/j.parkreldis.2017.10.012

23. Beetz C, Pieber TR, Hertel N, Schabhuttl M, Fischer C, Trajanoski S, et al. Exome sequencing identifies a REEP1 mutation involved in distal hereditary motor neuropathy type V. Am J Hum Genet. 2012;91(1):139-45. doi:10.1016/j.ajhg.2012.05.007

24. Richard S, Lavie J, Banneau G, Voirand N, Lavandier K, Debouverie M. Hereditary spastic paraplegia due to a novel mutation of the REEP1 gene: Case report and literature review. Medicine. 2017;96(3):e5911. doi: 10.1097/MD.000000000000591

**7** **Supplementary Figure**


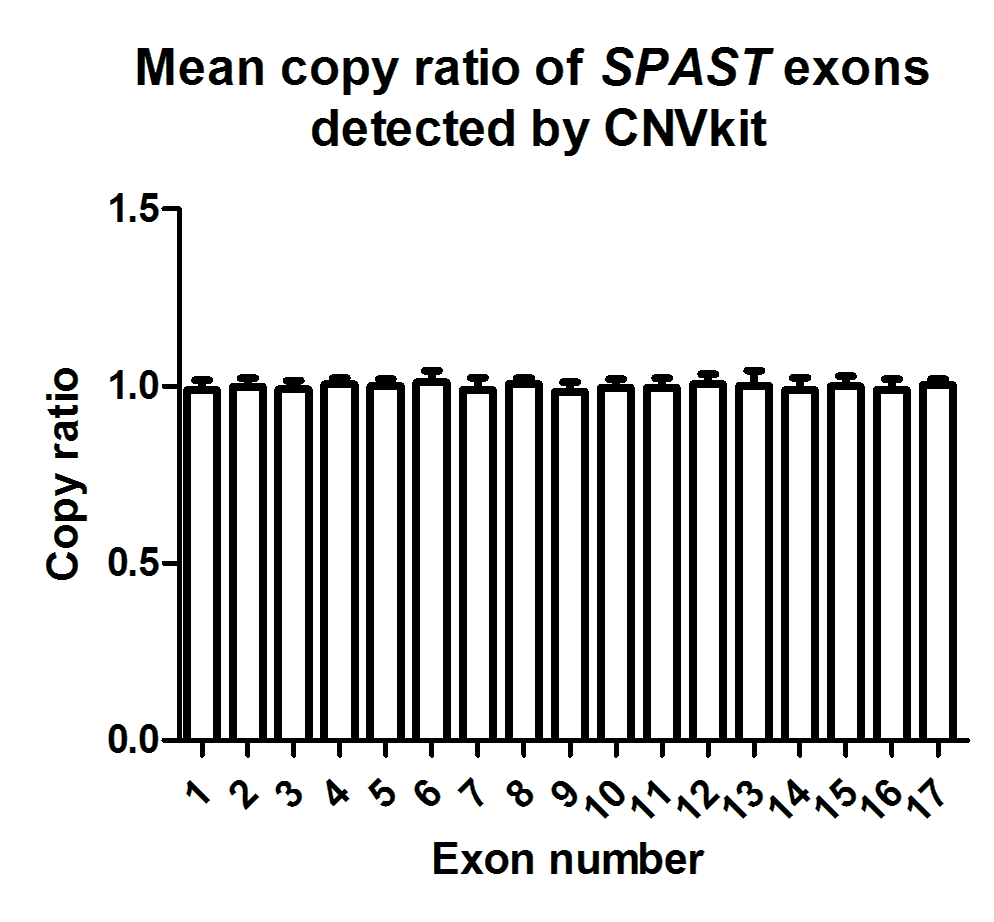


**sFigure 1.** Mean copy ratio of *SPAST* exons detected in NGS data by CNVkit. Error bars showed standard error. Three copy number analyzing tools were used. No abnormal copy number variants in *SPAST* were detected by all 3 tools.


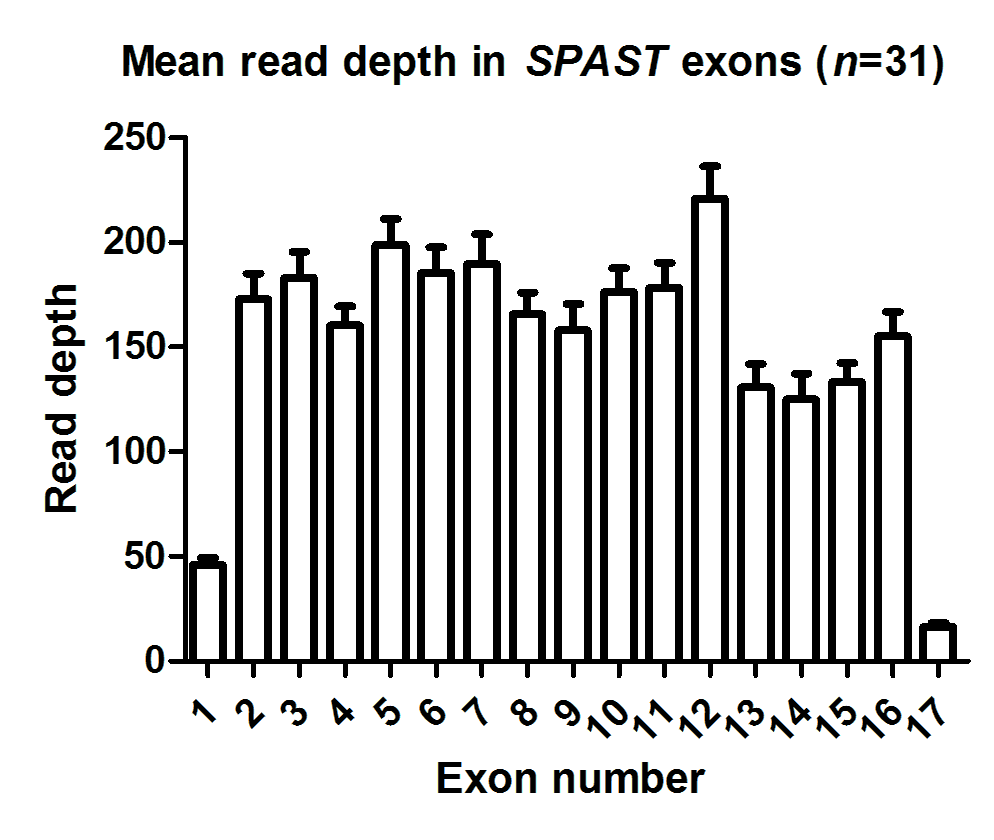


**sFigure 2.** Mean read depth in *SPAST* exons. Error bars showed standard error. The first exon and 17th exon include untranslated regions that can't be captured by probes.
